# Contextual Fear Memory Maintenance Changes Expression of pMAPK, BDNF and IBA-1 in the Pre-limbic Cortex in a Layer-Specific Manner

**DOI:** 10.3389/fncir.2021.660199

**Published:** 2021-07-06

**Authors:** Nicholas Chaaya, Joshua Wang, Angela Jacques, Kate Beecher, Michael Chaaya, Andrew Raymond Battle, Luke R. Johnson, Fatemeh Chehrehasa, Arnauld Belmer, Selena E. Bartlett

**Affiliations:** ^1^School of Clinical Sciences, Faculty of Health, Queensland University of Technology, Brisbane, QLD, Australia; ^2^Translational Research Institute, Faculty of Health, Queensland University of Technology, Brisbane, QLD, Australia; ^3^School of Biomedical Science, Faculty of Health, Queensland University of Technology, Brisbane, QLD, Australia; ^4^University of Queensland Diamantina Institute, Translational Research Institute, Brisbane, QLD, Australia; ^5^School of Psychology and Counselling, Faculty of Health, Queensland University of Technology, Brisbane, QLD, Australia; ^6^Center for the Study of Traumatic Stress, Department of Psychiatry, USU School of Medicine, Bethesda, MD, United States

**Keywords:** fear, learning and memory, plasticity, prelimbic cortex, brain derived neurotrophic factor (BDNF)

## Abstract

Post-traumatic stress disorder (PTSD) is a debilitating and chronic fear-based disorder. Pavlovian fear conditioning protocols have long been utilised to manipulate and study these fear-based disorders. Contextual fear conditioning (CFC) is a particular Pavlovian conditioning procedure that pairs fear with a particular context. Studies on the neural mechanisms underlying the development of contextual fear memories have identified the medial prefrontal cortex (mPFC), or more specifically, the pre-limbic cortex (PL) of the mPFC as essential for the expression of contextual fear. Despite this, little research has explored the role of the PL in contextual fear memory maintenance or examined the role of neuronal mitogen-activated protein kinase (pMAPK; ERK 1/2), brain-derived neurotrophic factor (BDNF), and IBA-1 in microglia in the PL as a function of Pavlovian fear conditioning. The current study was designed to evaluate how the maintenance of two different long-term contextual fear memories leads to changes in the number of immune-positive cells for two well-known markers of neural activity (phosphorylation of MAPK and BDNF) and microglia (IBA-1). Therefore, the current experiment is designed to assess the number of immune-positive pMAPK and BDNF cells, microglial number, and morphology in the PL following CFC. Specifically, 2 weeks following conditioning, pMAPK, BDNF, and microglia number and morphology were evaluated using well-validated antibodies and immunohistochemistry (*n* = 12 rats per group). A standard CFC protocol applied to rats led to increases in pMAPK, BDNF expression and microglia number as compared to control conditions. Rats in the unpaired fear conditioning (UFC) procedure, despite having equivalent levels of fear to context, did not have any change in pMAPK, BDNF expression and microglia number in the PL compared to the control conditions. These data suggest that alterations in the expression of pMAPK, BDNF, and microglia in the PL can occur for up to 2 weeks following CFC. Together the data suggest that MAPK, BDNF, and microglia within the PL of the mPFC may play a role in contextual fear memory maintenance.

## Introduction

Post-traumatic stress disorder (PTSD) is a debilitating and enduring fear-based disorder, typically associated with many cycles of relapse (Boschen et al., 2009; Maren, 2011). The contextual component of fear allows for the long-term storage of fear memories and can prevent the extinction of such memories (Maren, 2011). In animals, such as rodents, this contextual component can be manipulated and studied via the use of Pavlovian fear conditioning protocols (Foa et al., 1992; Rothbaum and Davis, 2003; Fanselow, 2010; LeDoux, 2014; Chaaya et al., 2018). Contextual fear conditioning (CFC) is a particular Pavlovian conditioning procedure that occurs when rodents are placed into a context (conditioned stimulus, CS) and provided with noxious stimuli (unconditioned stimulus, US; Foa et al., 1992; Rothbaum and Davis, 2003; Fanselow, 2010; LeDoux, 2014; Chaaya et al., 2018). Extensive investigations into the anatomical alterations following CFC have identified the amygdala and dorsal hippocampus (DH) as two brain regions essential for its acquisition, consolidation, expression, and maintenance (reviewed extensively in Chaaya et al., 2018). However, additional research has identified numerous other brain regions to be involved in CFC, one being the medial prefrontal cortex (mPFC; Gilmartin et al., 2014; Rozeske et al., 2015).

Interestingly, various studies have demonstrated damage or interference to the mPFC prior to CFC to have little effect on its consolidation (Morgan et al., 1993; Morrow et al., 1999a; Fernandez Espejo, 2003; Antoniadis and McDonald, 2006; Bissière et al., 2008). However, evidence of mPFC, or more specifically pre-limbic cortex (PL), involvement in the expression of fear-related freezing behaviour to context is well documented (Frankland et al., 2004; Corcoran and Quirk, 2007; Laurent and Westbrook, 2008; Goshen et al., 2011; Stevenson, 2011; Stern et al., 2013). A particular constraint of these studies, however, is that they only evaluate contextual fear memories soon after conditioning; they do not provide data into the long-term maintenance of contextual fear memories. Therefore, the current study examines the alterations that occur in the PL 2 weeks following CFC. In order to study fear maintenance and not expression, conditioned rodents are sacrificed without memory recall (fear expression brain circuits remain inactive).

Various alternate conditioning procedures can lead to the development of contextual fear memories. One such procedure is the unpaired fear conditioning (UFC) protocol (Trifilieff et al., 2007; Bergstrom et al., 2011, 2013). UFC is fundamentally identical with CFC, in that rodents are placed into a context and provided with noxious stimuli. The main alteration of this protocol is the presentation of random and non-overlapping stimuli (typically, auditory tones). Despite the introduction of auditory tones, fear remains specific to context; with no noticeable changes in fear to tone present (Bergstrom et al., 2011, 2013; Chaaya et al., 2019). This protocol also has similarities to cued fear conditioning (e.g., auditory fear conditioning; AFC). During AFC, the presentation of auditory tones and noxious stimuli overlap, forcing the contextual component to become a “background” stimulus (Phillips and LeDoux, 1994). This creates fears to tone (as well as “background” fear to context”). As a result of the similarities between these protocols, yet the difference in fear memory formation to tone, UFC has typically been used as a control for AFC (McKernan and Shinnick-Gallagher, 1997; Rogan et al., 1997; Majak and Pitkänen, 2003; Radley et al., 2006; Bergstrom et al., 2011, 2013). On numerous occasions, research has shown that the explicit pairing of auditory tone and noxious stimuli (AFC) results in greater activation of the amygdala as compared to the un-pairing (UFC) of these auditory tones and noxious (McKernan and Shinnick-Gallagher, 1997; Rogan et al., 1997; Majak and Pitkänen, 2003; Radley et al., 2006; Bergstrom et al., 2011, 2013). However, the development of contextual fear memories following UFC suggests that the amygdala may also be recruited. Unsurprisingly, this has been demonstrated previously (Trifilieff et al., 2007). Additionally, research from our lab (Chaaya et al., 2019) recently demonstrated an increase in immediate early gene expression in the lateral amygdala (LA) 90 min after UFC. The current study aims to expand on these findings and determines if similar changes in PL are present 2 weeks following UFC.

Neuromodulatory changes in the PL following fear conditioning may result from several molecular processes. Brain derived neurotrophic factor (BDNF) is secreted within the cerebral cortex (Lin et al., 1998) where it induces a variety of neuroplastic events. The hippocampal secretion of BDNF mediates neural activity associated with memory formation and storage (Cirulli et al., 2004; Bekinschtein et al., 2007; Heldt et al., 2007), including the long-term storage of fear memories (Lubin et al., 2008). Additionally, pharmacological activation of BDNF”s cognate receptor, TrkB, prevents defects in fear memory in aged rats (Zeng et al., 2012). However, the influence of BDNF on fear memory formation in other brain regions involved in fear memory maintenance, such as the PL cortex, is unknown. Another marker of neuronal activity, mitogen-activated protein kinase (MAPK/ERK1/2; Sweatt, 2004) is phosphorylated at the 42nd and 44th residues (pMAPK) as a result of fear conditioning (Bergstrom et al., 2011). In addition, MAPK phosphorylation driven by BDNF signalling has been shown to enhance fear (Revest et al., 2014). However, these findings are once again confined to the hippocampus; the importance of PL BDNF and pMAPK in the long-term storage of fear memories is not known.

In addition to BDNF and MAPK, microglia also facilitate neuromodulation (Walker et al., 2014; Wu et al., 2015; Dwyer and Ross, 2016; Wohleb, 2016). Their ability to modulate long-term fear memories and influence PTSD is well documented (Enomoto and Kato, 2021). Microglia respond to CNS insult or injury in two ways: the total number of microglia in the affected area increases and the morphological characteristics of these microglia change (Kettenmann et al., 2011; Calcia et al., 2016; Dwyer and Ross, 2016). Resting microglia in the healthy CNS, referred to as “ramified”, have long thin extensions that actively search for signals of insult (Kettenmann et al., 2011; Calcia et al., 2016; Dwyer and Ross, 2016). Upon detection of insult or injury, microglia number increases, and morphology change to become “amoeboid”. This amoeboid shape involves a larger cell body size accompanied by a reduction in branching and number of extensions (Kettenmann et al., 2011; Walker et al., 2014; Dwyer and Ross, 2016). We have previously shown alterations in hippocampal microglia number and morphology in response to CFC (Chaaya et al., 2019). However, no research to date has examined the role of microglia, BDNF, and pMAPK within the PL in Pavlovian fear conditioning. Therefore, the current experiment is designed to assess microglial number and morphology, as well as BDNF and pMAPK expression in the PL following CFC, UFC, and a context only (CO) control.

## Materials and Methods

### Animals

Experimentally naïve male Sprague Dawley Rats (Animal Resources Centre, WA, Australia) weighing 176–200 g at the time of arrival were housed, two per cage, by the University of Queensland Biological Resources (UQBR) facility. Rats were maintained on a 12-h light/dark cycle, with food provided *ad libitum*. Behavioural procedures were approved by the University of Queensland (Ethics approval no. 460/17) and the Queensland University of Technology (QUT number: 80087) animal ethics unit. Behavioural procedures complied with the Queensland Government Animal Research Act 2001, associated Animal Care of Animals for Scientific Purposes, 8th Edition (National Health and Medical Research Council, 2013) policies and regulations of animal experimentation and other ethical matters. Upon delivery, rats were left to acclimatise to the UQBR Facility for 8 days. The experimenter handled all rats for 9 days, habituated them to the fear conditioning chamber for 1 day, and then fear-conditioned them on the next day. All rats were then returned to their home-cages for 2 weeks. Three separate groups existed: two experimental (Contextual Fear Conditioned, CFC *n* = 18 and Unpaired Fear Conditioned, UFC, *n* = 18) and one control (Context Only, CO *n* = 18). Two weeks after conditioning rats were divided into an anatomical (*n* = 12 per group) subgroup or behavioural (*n* = 6 per group) subgroup.

### Conditioning

Two Plexiglas conditioning chambers (Coulbourn Instruments, Lehigh Valley, PA, USA) were used for fear conditioning procedures (context A and B). Both context A and B were sound insulated (background dB = 55) and equipped with an infrared camera and speaker. Context A was fitted with a metal grid floor that was attached to an electric shock generator. Context A had no decorations and, following the presentation of each rat, was cleaned with ethanol (EtOH) 80%. Context B had a flat floor that was lightly covered with bedding. The roof was altered so its physical dimensions differed from context A. The walls were coloured and orange-scented hand soap was used to clean the chamber after each rat. The bedding was also replaced. One day following handling, all rats were placed in context A for a 30 min habituation session. One day after habituation, rats in the CFC and UFC group were placed back into context A for fear-conditioning. Rats in the CO control group were placed in context A without any added stimuli. Fear conditioning for rats in the CFC group consisted of a 180 s exploration period whilst in the context, followed by the presentation of five non-overlapping and random electric foot-shocks (1.0 mA, 0.50 s). Rats were left for 60 s following the presentation of the final foot-shock, and then immediately returned to their home-cages. Fear conditioning for rats in the UFC group was the same as for rats in the CFC group, except with the addition of five non-overlapping (with each other or the foot-shocks) and random auditory tones (5 kHz, 75 dB, 20 s). In total, conditioning procedures were 660 s long for rats in the CFC and CO control groups and 880 s for rats in the UFC group (extra time was required to account for the auditory tones). Freezing behaviour was scored during fear conditioning to provide a measure of progressive fear-learning. Freezing behaviour was scored before (baseline), during (cue 1–5), and after fear conditioning (final).

Upon return to home-cages, rats were left undisturbed for 2 weeks. The process of memory storage and maintenance is hypothesised to involve the restructuring and reorganisation of brain regions in order to support the permanent storage of a memory (Dudai, 2004). This process is hypothesised to last for as little as several days to as long as several months (Dudai, 2004; Rodrigues et al., 2004; Frankland and Bontempi, 2005; Kandel et al., 2014). The current study examined changes that occurred ins the 2-week time-frame in order to ensure the memory storage/maintenance phase is captured (Dudai, 2004).

### Behavioural Procedures

**Figure 1:**
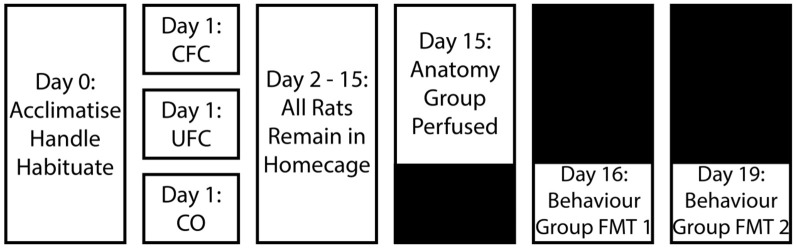
Experimental design for behavioural training. Following a period of acclimatisation to the vivarium, handling and habituation to the fear conditioning context, rats were divided into three behavioural groups. Rats in the occupying context A were provided with five non-overlapping and random electric shocks to the foot (CFC group). Alternatively, another group of rats in context A were provided with the same foot-shocks, as well as five non-overlapping and random auditory tones (UFC group). The final group of rats (CO control group) were placed into context A and left undisturbed without the addition of any stimuli. After conditioning, all rats were returned to their home cages and left undisturbed for 2 weeks. Rats were further sub-divided into a behavioural (provided with two fear memory tests (FMT) separated by 3 days) and anatomical (perfused 2 weeks post-conditioning) group. CFC, Contextual fear conditioning; UFC, unpaired fear conditioning; CO, context only.

Behavioural procedures are outlined in Figure 1 and are discussed below.

Two weeks following conditioning procedures, the behavioural subgroup of rats (*n* = 6 per group) were provided with two fear memory tests (FMT) to assess freezing behaviour. During the first FMT (FMT to context), rats were placed back into context A (where conditioning occurred) for 10 min, whereby freezing behaviour was manually scored. No foot-shock or auditory tones were provided. Three days following, rats were provided with a second FMT (FMT to tone). During this FMT, rats were placed into a new context (context B) for 10 min and presented with 10 auditory tones (5 kHz, 75 dB, 20 s). As conducted previously (Bergstrom et al., 2011, 2013; Chaaya et al., 2019; Jacques et al., 2019), these auditory tones were presented during the final 20 s of each minute.

Freezing behaviour, defined as the inhibition, absence or suppression of movement, was scored in 20 s blocks during training and testing (Phillips and LeDoux, 1992, 1994; Quirk et al., 1997; Radley et al., 2006; Bergstrom et al., 2013; Bergstrom and Johnson, 2014). The movement required for autonomic nervous system function (e.g., heavy breathing and minimal movement) was included as freezing, whereas head scanning and sleeping were not (Fanselow, 2010). Freezing behaviour during CFC and UFC training was scored in the final 20 s of the first minute (baseline), the final 20 s of the last minute (final) and the 20 s prior to each foot-shock (cue 1–5). As the current experimental design does not utilise an auditory cue fear conditioned group, the word “cue” as seen in Figure 3 refers to foot-shock presentations. An exception is made for rats in the CO control group, whereby freezing behaviour was scored at identical time points as the CFC group (but still referred to as cue 1–5). For rats in the UFC group, freezing behaviour was not scored in the 20 s prior to each auditory tone, as the development of contextual fear memories is of interest here. During testing, freezing was scored in the final 20 s of each minute (Bergstrom et al., 2011, 2013).

**Figure 2:**
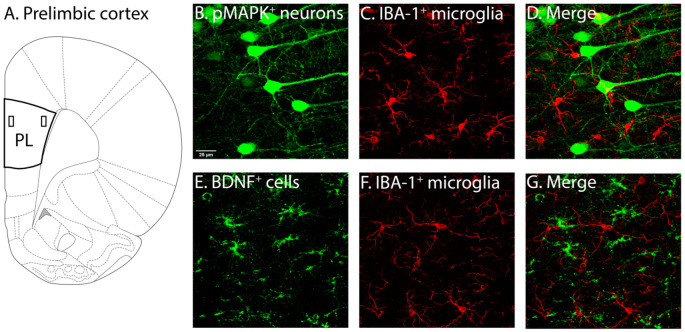
Illustration of PL and associated labelling in this region. **(A)** Two layers of the PL were of interest, layers 2–3 (demarcated by the rectangle closer to the border) and layers 5–6 (demarcated by the rectangle further from the border). Following identification of PL layers 2–3 and 5–6 (Miner et al., 2003; Perez-Cruz et al., 2007) used as guides, pMAPK expressing neurons **(B)**, BDNF expressing cells **(E)** and IBA-1 **(C–F)** labelled microglia, as well as their co-localisation **(D–G)** were quantified. PL, pre-limbic cortex; pMAPK, phosphorylated mitogen-activated protein kinase; BDNF, brain-derived neurotrophic factor; IBA-1, ionized calcium-binding adaptor molecule 1.

**Figure 3:**
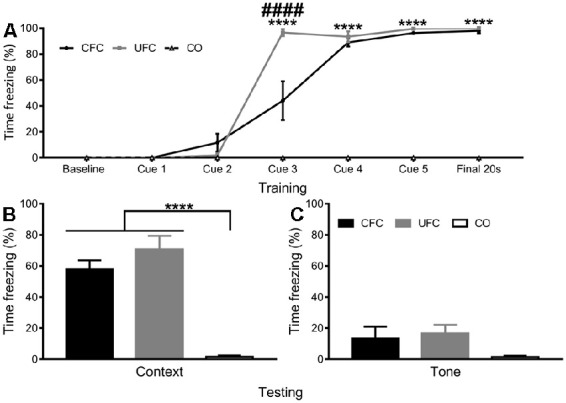
Freezing to Context and Tone Data. **(A)** To obtain a progressive measure of fear learning, freezing behaviour was scored during training. Scoring was scored in the final 20 s of the first minute, the 20 s prior to each foot-shock, and the final 20 s of the final minute. Analyses revealed rats in the CO control to consistently have no fear-related freezing to context, whereas rats in the CFC and UFC groups progressively developed fear memories to context. As seen, by cue 3, rats in the CFC and UFC had significantly more fear-related freezing as compared to the CO control. Rats in the UFC also had significantly more fear-related freezing to context as compared to the CFC group. However, by cue 4, cue 5 and final 20 s, this had equalized, with both CFC and UFC groups have equivalent levels of freezing behaviour, which was significantly higher than that in rats in the CO control group. Asterisks denote level of statistical significance between both CFC and UFC vs. the CO control ****p ≤ 0.0001. Hash symbol denote level of statistical significance between the UFC and CO control group ^####^*p* ≤ 0.0001. **(B)** Two weeks following conditioning, rodents were re-exposed to context A, and a FMT was conducted. As seen, rats in both the CFC and UFC groups froze significantly more than rats in the CO control group. **(C)** To ensure that no additional fear to tone developed in rats in the UFC, 3 days following the first FMT, all rats were exposed to the new context B and provided with auditory tones. Statistical analyses revealed no statistically significant increases in either of the fear-conditioned groups. CFC, contextual fear conditioning; UFC, unpaired fear conditioning; CO, context only; FMT, fear memory test. Asterisks denote level of statistical significance between groups ****p ≤ 0.0001.

### Immunohistochemistry

Two weeks following conditioning procedures, rats in the anatomical subgroup (*n* = 12) were perfused. To ensure memory reactivation/reconsolidation did not occur, rats were anaesthetised in a room separate from where fear conditioning occurred. This room was located on a separate corridor to the fear conditioning room. Rats had not been previously exposed to this room.

Rats in the anatomical group (*n* = 12 per group) were provided with intraperitoneal (i.p.) injections of a ketamine/xylazine cocktail (100 mg/kg, 10 mg/kg respectively). Following anaesthetisation, ice-cold saline (200 ml per rat), followed by 4% paraformaldehyde/0.1 M phosphate buffer (PB; pH of 7.4; 400 ml per rat) were used to transcardially perfuse the rats. Brains were subsequently removed and stored for 24 h at 4°C in the 4% paraformaldehyde fixative. Following, brains were washed with phosphate buffered saline (PBS) and then stored in PBS/0.02% Azide for a minimum of 3 days. A vibratome (M11000; Pelco easiSlicer, Ted Pella Inc, CA, USA) was used to obtain sequential, free-floating, 40 μm thick coronal brain sections containing the mPFC. Sections were stored at 4°C in PBS/0.02% Azide until immunohistochemistry procedures commenced.

Immunohistochemistry was conducted on the left hemisphere sections. Two sections (equal distances apart; approximately 30 μm) per antibody combination were taken from each rat. For example, from a single rat, two sections (approximate Bregma coordinate + 3.00 mm and + 2.76) were labelled with pMAPK/IBA-1 (24 per group), and two sections (approximate Bregma coordinate + 2.96 mm and + 2.72 mm) labelled with BDNF/IBA-1 (24 per group).

Stored brain sections were removed from PBS/0.02% Azide and washed with PBS. Two separate immunohistochemistry protocols were required for double-labelling of pMAPK/IBA-1 and BDNF/IBA-1. All sections were permeabilised with 1% Triton/0.1% Tween 20 in PBS for 1 h and then washed in PBS. Only sections labelled for BDNF/IBA-1 were incubated in Citrate Buffer (10 mM Sodium Citrate, 0.05% Tween 20, pH 6.0) for 5 min (80^o^C). Once these sections returned to room temperature, they were washed with PBS. All sections (pMAPK/IBA-1 and BDNF/IBA-1) were then incubated in blocking solution 0.3% Triton/0.5% Tween 20/2% NHS in PBS/Azide 0.02% for 1 h.

Sections labelled for pMAPK/IBA-1 were removed from the blocking solution and immediately incubated in an anti-IBA-1 (ab5076) goat polyclonal antibody (1:500; Abcam, VIC, Australia) diluted in the blocking solution for 48 h. Following, these sections were washed in the blocking solution, and then immediately incubated in a cross-adsorbed donkey anti-goat IgG (H + L; Alexa Fluor 594) secondary antibody (1:500; ThermoFisher Scientific, VIC, Australia) in a blocking solution for 3 h. These sections were then washed in the blocking solution, PBS, and then a new blocking solution that did not contain Triton. Brain sections were subsequently incubated in a phosphor-p44/42 MAPK antibody (Erk 1/2; Thr 202/Tyr 204; 1:150; #4370, Cell Signalling Technology, MA, USA) diluted in this new blocking solution for 48 h. Following this, brain sections were washed in the new blocking solution, incubated in a cross-adsorbed donkey anti-rabbit IgG (H + L; Alexa Fluor 488) secondary antibody (1:500; ThermoFisher Scientific, VIV, Australia) in the new blocking solution for 3 h, washed again in this blocking solution, washed in PBS, incubated in 4′, 6-diamidino-2-phenylindole (DAPI; D1306) diluted in PBS for 5 min (1:1,000 ThermoFisher Scientific, VIC, Australia), washed in PBS, and then mounted on silane coated slides. Mounted sections were cover-slipped immediately, left to dry, and stored at 4^o^C.

Sections labelled for BDNF/IBA-1 were removed from the blocking solution and immediately incubated in the anti-IBA-1 antibody and an anti-BDNF [EPR1292] (ab108319) rabbit monoclonal antibody (1:500; Abcam, VIC, Australia) for 48 h. The anti-pMAPK antibody (RRID: AB_2315112), anti-IBA-1 antibody (Monasor et al., 2020; RRID: AB_2224402; and the anti-BDNF antibody; Gideons et al., 2017; RRID: AB_10862052) have been previously validated (pMAPK) or used in immunohistochemistry (IBA-1 and BDNF). These antibodies were diluted in the previous blocking solution without triton X100. Following the 48 h incubation period, sections were washed in the blocking solution without triton, and then immediately incubated in the donkey anti-rabbit (488) and donkey anti-goat (594) secondary antibodies for 3 h. Sections were immediately washed in blocking solution and then PBS before being incubated in DAPI for 5 min. Finally, these sections were washed in PBS and then mounted and cover-slipped similar to the pMAPK/IBA-1 sections. All antibodies were validated.

### Imaging and Analysis

Cover-slipped sections labelled for pMAPK/IBA-1 and BDNF/IBA-1 were scanned using the Olympus FV3000 Confocal Laser Scanning Microscope (Olympus Australia Pty, Ltd, VIC, Australia). A sample of approximately layers 2–3 and layers 5–6 (see Figure 2) of the PL were obtained with 40x magnified (1.5x zoom) scans (*x* = 212.132 μm, *y* = 413.699 μm) with 30 *z*-stacks of 0.50 μm thickness (*z* = 15 μm) in the 488 nm and 561 nm channel.

Neurons and microglia were semi-automatically counted and tagged with the “spots” tool in IMARIS (IMARIS 9.1.2, Bitplane). The experimenter was made blind to the coding of the datasets. The average diameter of cells labelled with pMAPK, BDNF and IBA-1 was measured. Cells had to have clearly identifiable cell bodies, with at least one section visible to be counted (see Figure 2). Filter intensity was altered to ensure all cells expressing pMAPK, BDNF and IBA-1 were accurately tagged. The incorrectly tagged artefact was manually removed by the experimenter, and untagged labelled cells manually added. The number of IBA-1, pMAPK, and BDNF expressing neurons was then measured.

Morphological differences in microglia were examined if the total number of IBA-1 cells was different as a function of fear conditioning. A total of 24 microglia were traced per group using Neurolucida 360 (Neurolucida 360, MBF Bioscience, VT, USA). The average length of the microglia extensions, number of trees or ends of these extensions and complexity of the microglia branching (measure of ramification state) were quantified from these traces. The following formula was utilised to determine the complexity of microglia branching (sum of the terminal orders + the number of terminals) * (total process length/number of primary branches) (Pillai et al., 2012).

### Data Analysis

The results section is separated into a behavioural and anatomical section. Within the anatomical section, results are separated depending on the PL layer: PL 2–3 and PL 5–6. Within each part, differences as a function of conditioning (CFC, UFC and CO control) across the two PL layers are explored. All differences were explored with analyses of variances (ANOVA). Prior to analyses, normality and homogeneity of variance were tested for. Due to the large dataset, breaches in normality and homogeneity could be identified. These breaches may result in the inflation of type I errors. D’Agostino and Pearson normality test was performed to assess the normality of the data. All data analysed were deemed normal (alpha > 0.05). All values in the text and graphs are expressed + /− standard error of the mean. Statistical significance is identified with *p* values at or below 0.05. Statistical analyses were conducted using GraphPad Prism v8 software (GraphPad, CA, USA). Asterisks within the graphs denote level of statistical significance (*p ≤ 0.05; **p ≤ 0.01; ***p ≤ 0.001; ****p ≤ 0.0001).

### Excluded Cases

Cases were excluded from analyses for various reasons. Prior to analyses, sections that were significantly damaged through the regions of interest, or that failed to label during immunohistochemistry procedures, were excluded. Furthermore, statistical outliers, identified via the ROUT method (GraphPad Prism), with the maximum false discovery rate set to 1%, were excluded on a pairwise basis. The ROUT method identifies multiple outliers in large datasets, making it appropriate for the data here. Outlier analyses were conducted on individual groups. Outliers and damaged/failed sections were excluded on a pairwise basis.

For FMT1, 1 outlier was removed from cues 4 and 5 in UFC, 1 outlier was removed from cue 4 in CFC and 1 outlier was removed from cue 9 in CO. For FMT2, 1 outlier was removed from cues 1, 3 and 9 in UFC, 1 outlier was removed from cues 2, 3, 4, 8 and 9 in CFC. For anatomical analysis, both UFC and CFC had 2 outlier/damaged samples removed each. The sample size did not fall below *n* = 9 (per group) for all anatomical analyses or *n* = 5 for behavioural analyses.

## Results

### Behavioural Results

To confirm that the fear conditioning procedures produced long-term behavioural alterations, the number of time rats displayed freezing behaviour during training and testing was quantified as a function of condition. During training, mean freezing (provided as a percentage) across behavioural conditions was quantified during each time-point (baseline, cue 1, cue 2, cue 3, cue 4, cue 5, and final). A two-way mixed-design ANOVA was used to analyse changes as a function of time-point (within-subjects factor) and condition (between-subjects factor). Analyses revealed a statistically significant interaction (*F*_[12, 100]_ = 53.1588, *p* < 0.0001) of freezing behaviour as a function of time-point and condition, as well as a significant main-effect of time-point (*F*_[6, 100]_ = 186.9417, *p* < 0.0001) and a significant main effect of condition (*F*_[2, 100]_ = 397.3932, *p* < 0.0001). Bonferroni-corrected *post hoc* tests (see Figure 3A) followed up simple main-effects of condition at each time-point. Analyses reveal no group differences to exist at the baseline, cue 1, or cue 2 time-points. At cue 3, significant differences exist between the CFC (freezing response was 44.05%) and CO control (0%), UFC (96.62%) and CO control and CFC and UFC groups. By cue 4, rats in the CFC (88.98%) and UFC (93.62%) group had statistically equivalent fear, which were both significantly higher than the CO control (0%). This pattern continued for cue 5 and the final time-point, indicating progressive development of fear to the context in the conditioned rats, with no development of random fear in the CO control group (Figure 3A).

Two weeks following training, FMT to context and FMT to tone (3 days later) were provided to ensure long-term fear memories had developed. Freezing behaviour was compared as a function of behavioural condition. One-way ANOVA during the FMT to context revealed significant differences to exist between groups (*F*_[2, 15]_ = 35.8949, *p* < 0.0001). Bonferroni-corrected *post hoc* tests revealed rats in the CFC (57.80%) group to have significantly more freezing behaviour than the rats in the CO control (1.67%) group (Figure 3B). Similarly, rats in the UFC (70.65%) group expressed significantly more freezing behaviour than rats in the CO control group (Figure 3B). There were no statistical differences in freezing behaviour between the CFC and UFC group. To ensure fear to tone did not develop during the UFC protocol, an FMT to tone was conducted 3 days later. Freezing behaviour was measured during auditory tone presentation in a new context. One-way ANOVA revealed no significant group differences to exist as a function of behavioural condition (*F*_[2, 15]_ = 2.0677, *p* = 0.1610; Figure 3C).

### Contextual Fear Maintenance Increases the Expression of BDNF, Neuronal pMAPK and Microglial IBA-1 in Layers 2–3 of the Prelimbic Cortex

**Figure 4:**
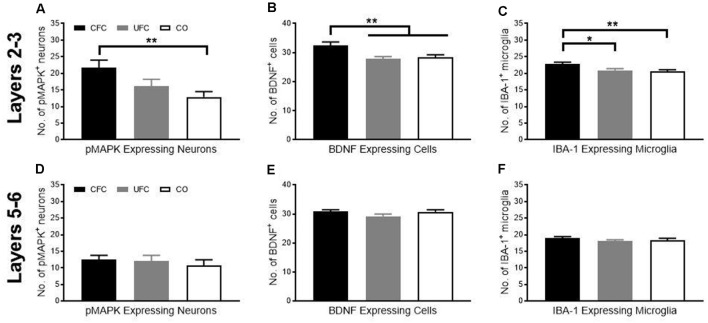
pMAPK, BDNF and IBA-1 number in PL following CFC and UFC. Evaluation of pMAPK expressing neurons **(A)** in PL layer 2–3 revealed significant group differences to exist as a function of condition. Rats that underwent CFC had significantly more pMAPK expressing neurons as compared to rats in the CO control group. No further group differences were found. Similarly, evaluation of BDNF expressing cells **(B)** and IBA-1 labelled microglia **(C)** revealed group differences to exist as a function of condition. In both cases, rats in the CFC group had significantly more BDNF and IBA-1 labelled cells than both the CO control and UFC groups. In PL layer 5–6, rats that underwent CFC or UFC show no difference in **(D)** the number of pMAPK expressing neurons, **(E)** BDNF expressing cells and **(F)** IBA-1 labelled cells. PL, pre-limbic cortex; pMAPK, phosphorylated mitogen-activated protein kinase; BDNF, brain derive neurotrophic factor; IBA-1, ionized calcium-binding adaptor molecule 1; CFC, contextual fear conditioning; UFC, unpaired fear conditioning; CO, context only. Asterisks denote level of statistical significance between groups *p ≤ 0.05; **p ≤ 0.01.

Although the role of BDNF, MAPK phosphorylation, and microglia in fear memory storage is well document in the hippocampus, the importance of prelimbic BDNF, pMAPK, and microglia for the maintenance of fear memory is unknown. We, therefore, measured changes in expression of pMAPK, BDNF, and IBA-1 in approximate layers 2–3 of the PL as a function of behavioural condition. We then calculated the number of cells expressing pMAPK, BDNF, and IBA-1 as a function of condition. One-way ANOVA revealed a significant group difference (*F*_[2, 59]_ = 5.0052, *p* < 0.01) in the number of pMAPK expressing neurons. Bonferroni-corrected* post hoc* tests showed that rats in the CFC group had significantly more pMAPK expressing neurons as compared to the CO control group (Figure 4A). Similarly, one-way ANOVA revealed a significant group difference (*F*_[2, 69]_ = 7.1616, *p* < 0.01) in the number of BDNF expressing cells. Bonferroni-corrected* post hoc* tests revealed rats in the CFC to have significantly more BDNF expressing cells than the CO control (Figure 4B). Interestingly, BDNF expression was also higher in the CFC group as compared to the UFC group. Finally, one-way ANOVA of IBA-1 expression revealed a significant group difference (*F*_[2, 131]_ = 6.3744, *p* < 0.01) in the number of IBA-1 expressing cells. Similar to BDNF expressing cells, IBA-1 microglia were significantly higher in the CFC group as compared to both the CO control and UFC group (Figure 4C). This suggests that layers 2 and 3 of the prelimbic cortex are sensitive to the long-term impact of fear memories.

**Figure 5:**
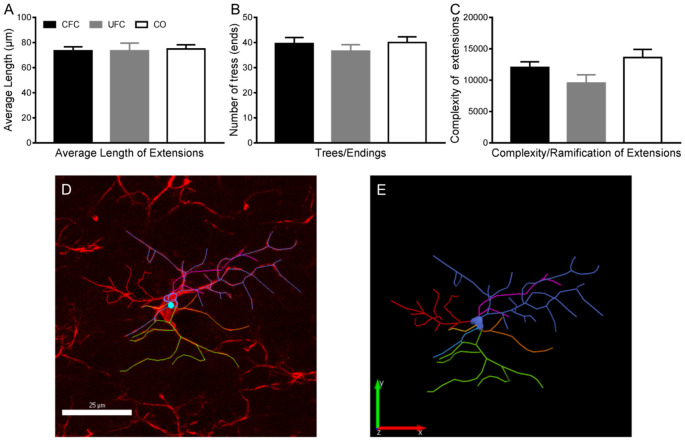
Microglia morphology in PL layer 2–3. Evaluation of microglia morphology in PL layer 2–3 revealed no changes as a function of condition. As seen, the average length of extensions **(A)**, number of trees/endings **(B)** and complexity of ramifications **(C)** remained statistically similar in all three behavioural groups. **(D)** Representative traces microglia with tracing overlaid, and **(E)** the tracing alone. PL, pre-limbic cortex. Scale bar = 25 μm.

Given that the morphology of microglia is strongly indicative of their function in neuromodulation (Enomoto and Kato, 2021), we then examined whether, in addition to a change in cell number, there was also a change in the morphology following CFC. Morphological alterations of microglia were determined using three measures obtained from tracing the IBA-1 cells: (1) the average length of extensions, (2) the number of endings/trees of these extensions, and (3) the complexity (ramification state) of these extensions (Figures 5D–E). Despite a difference in the number of IBA-1, one-way ANOVA revealed no group differences in the average length of extensions (*F*_[2, 67]_ = 0.0349, *p* = 0.9657, Figure 5A), the number of endings (*F*_[2, 69]_ = 0.5806, *p* = 0.5623, Figure 5B), or the complexity of these extensions (*F*_[2, 64]_ = 2.956, *p* = 0.0592, Figure 5C). Together, these data suggest there are no morphological alterations to microglia 2 weeks following fear conditioning in layer 2–3 PL.

### Expression of BDNF, Neuronal pMAPK and Microglial IBA-1 in Layers 5–6 of the Prelimbic Cortex Are not Altered by Fear Conditioning

We examined the number of cells expressing pMAPK, BDNF, and IBA-1 in layers 5–6 of the PL as a function of condition as described for layers 2–3 of the PL. One-way ANOVA revealed no significant group difference in the number of pMAPK expressing neurons (*F*_[2, 57]_ = 0.3340, *p* = 0.7174; Figure 4D), BDNF expressing cells (*F*_[2, 69]_ = 1.6599, *p* = 0.1976; Figure 4E), or IBA-1 expressing microglia (*F*_[2, 130]_ = 0.8215, *p* = 0.4420; Figure 4F). Because there were no differences found in the expression of pMAPK, BDNF, or IBA-1, there were no further experiments conducted in layers 5–6 of the PL.

## Discussion

This study investigated the relative contribution of the PL in the mPFC in the long-term maintenance of contextual fear memories by examining changes in expression of BDNF, neuronal pMAPK, and microglial IBA-1. An evaluation of the freezing behaviour during training and testing demonstrated a progressive development of fear to the context in both the CFC and UFC groups. Interestingly, there is a discrepancy in the freezing behaviour seen at cue 3 for the UFC group, with a slightly greater amount of freezing detected compared to previous studies with identical protocols (Chaaya et al., 2019). Overall, we are unsure what has caused this discrepancy, however, it is likely due to an undefined acoustic variable given that the current CFC freezing behaviour results are identical to those obtained previously (Chaaya et al., 2019). For example, these experiments were conducted in different rooms within the animal facility, which may have altered the ability of the rats to detect the auditory cue, therefore precipitating greater freezing behaviour earlier in the test. The fear to context remained statistically higher compared to the CO group 2 weeks later in both the CFC and UFC groups. Importantly, no fear to tone developed for either group, suggesting the development of fear memories to context. While fear to context was equivalent in both conditioned groups, in rats that underwent CFC there were significant changes in layers 2–3, but not 5–6, of the PL in pMAPK expressing neurons, BDNF expressing cells and IBA-1 labelled microglia. Interestingly, despite alterations in IBA-1 number, there were not any changes to microglia morphology in any behavioural condition. These data suggest, even without recall or reactivation of fear, measurable changes in expression of pMAPK, BDNF and IBA-1 in layers 2–3 of the PL in the mPFC may continue for up to 2 weeks following CFC.

### CFC Increases the Number of pMAPK, BDNF and IBA-1 Expressing Cells

CFC, but not UFC, led to an increase in the phosphorylation of MAPK, as well as the expression of BDNF and IBA-1 in PL layers 2–3, but not layers 5–6. However, the expression of these markers remained unchanged in rats fear conditioned with an unpaired auditory stimulus. The contribution of the mPFC following CFC has been heavily documented (see recent reviews: Gilmartin et al., 2014; Rozeske et al., 2015), however less is known about the role of the PL layers 2–3. It is well known that the hippocampus and amygdala, are essential for contextual fear memory acquisition. In contrast, research studies on the mPFC suggest it plays a different role (Fanselow, 2010; Gilmartin et al., 2014; Rozeske et al., 2015). Studies have demonstrated that inhibition, damage, manipulation or removal of the mPFC prior to CFC to have no effect on subsequent fear-related freezing to context (Morgan et al., 1993; Morrow et al., 1999b; Fernandez Espejo, 2003; Antoniadis and McDonald, 2006; Bissière et al., 2008). However, the involvement of the mPFC, or more specifically, the PL, in the expression of these contextual fear memories is well documented (see review by Frankland et al., 2004; Corcoran and Quirk, 2007; Laurent and Westbrook, 2008; Goshen et al., 2011; Stevenson, 2011; Stern et al., 2013; Rozeske et al., 2015). These studies examined both short- and long-term contextual fear memory expression, generally demonstrating PL involvement in both (Rozeske et al., 2015). The results of the current experiments support these studies, further suggesting that the PL may be involved in contextual fear memory maintenance, as opposed to expression. Following CFC, we report increases in pMAPK expressing neurons, BDNF expressing cells and IBA-1 labelled microglia in the PL. Interestingly, these rats were fear conditioned and subsequently sacrificed without undergoing an FMT. That is, they did not have an opportunity to express fear. This particular experimental design allows us to conclude that the changes in PL are a result of contextual fear memory maintenance and not expression.

The mPFC is a multi-layered and complex structure with poorly defined boundaries (Santana and Artigas, 2017). The PL, located within the mPFC, contains six layers (Perez-Cruz et al., 2007; Santana and Artigas, 2017). These layers are 1, 2–3, 5–6 and 4, which is less clearly identified (Marek et al., 2013). While the PL is documented to be involved in fear consolidation, expression and extinction, little research has identified the contribution of individual layers during these processes (Marek et al., 2013; Rozeske et al., 2015). Those that have utilised auditory fear conditioning and extinction procedures, as opposed to the CFC procedures examined here. For example, previous research identified the connectivity of PL layers 5–6 to infralimbic cortex (IL) layers 5–6 to be essential for short–term auditory fear extinction (Marek et al., 2018). More recently, research from our lab showed short–term fear memory recall to increase plasticity exclusively in layers 2–3 (Jacques et al., 2019). While this study examined alterations in the mPFC immediately following auditory fear memory recall, the results appear to be similar to those of this study. In the current study, an increase in pMAPK and BDNF expression is seen in layers 2–3 of the PL following long-term CFC. Layers 2/3 of the PL are specifically sensitive to chronic pain-induced neuroplasticity (Mitrić et al., 2019), mediate the pathogenesis of major depressive disorder (Shrestha et al., 2015), and project to the basolateral amygdala (Vertes, 2004; Gabbott et al., 2005; Hirai et al., 2012; Song et al., 2015), area 35 of the perirhinal cortex (Hirai et al., 2012), and the nucleus accumbens (Gabbott et al., 2005). Area 35 of the perirhinal cortex gates long-term memory storage (Kajiwara and Tominaga, 2021), therefore the changes seen in layers 2/3 of the PL may be facilitating this memory formation through innervation of the perirhinal cortex. While additional research is required, we can preliminarily conclude that layers 2–3 of the PL may be involved in both long-term auditory and contextual fear memory storage/maintenance. The necessity of BDNF signalling in this storage/maintenance is yet to be determined and may be explored in the future by assessing CFC following the disruption of the BDNF-pMAPK signalling pathway. It is currently unknown if other brain regions show similar mechanisms of long-term maintenance following CFC, however, lesion studies suggest that the hippocampus may also influence the long-term storage of fear memories (Kim and Fanselow, 1992). Future investigations assessing similar molecular alterations in different brain regions are therefore warranted.

Data reported here demonstrate an enduring role for the PL up to 2 weeks following CFC, suggesting it may be the locus of long-term contextual fear memory storage. Leading hypotheses suggest two phases of memory consolidation: synaptic consolidation and systems consolidation (Frankland and Bontempi, 2005; Sah et al., 2008). Synaptic consolidation includes changes in neuronal structure to support memory formation. Alternatively, systems consolidation includes the restructuring of brain regions (Dudai, 2004). There are many theories of systems consolidation, namely the standard consolidation theory (SCT) and the multiple memory trace theory (MMTT; discussed heavily in these articles; Nadel and Moscovitch, 1997; Nader et al., 2000; Frankland and Bontempi, 2005; Chaaya et al., 2018). A commonality of both these theories is that as time progresses, newly formed memories that relied upon the medial temporal lobes (MTL) start to become reliant upon cortical brain regions (Chaaya et al., 2018). Data reported demonstrated that plasticity-related alterations (as measured with pMAPK and BDNF) are continuing to occur in the PL for up to 2 weeks after fear conditioning. Previous studies have demonstrated plasticity and activity-related changes to occur in the amygdala and hippocampus (see extensive review: Chaaya et al., 2018) immediately following CFC. Together, this suggests that contextual fear memories initially formed in the amygdala and hippocampus become stored and maintained in the PL.

Systems consolidation theories assert that memory formation begins in the MTL and eventually become more reliant upon cortical brain regions (Nadel and Moscovitch, 1997; Nader et al., 2000; Frankland and Bontempi, 2005; Chaaya et al., 2018). We highlight this cortical brain region to be the PL. However, prior research has shown similar changes in PL immediately following CFC (Zelikowsky et al., 2013). With the data reported here, it appears that the PL may have a permanent role in contextual fear memory acquisition and maintenance. Interestingly, these studies demonstrate a similar function for PL as the hippocampus in CFC. Contextual fear memories can form if either PL or hippocampus are damaged prior to, but not after, CFC (see reviews: Fanselow, 2010; Chaaya et al., 2018 and Morgan et al., 1993; Young et al., 1994; Morrow et al., 1999a, b; Rudy et al., 2002; Fernandez Espejo, 2003; Antoniadis and McDonald, 2006; Bissière et al., 2008). Interestingly, previous research has shown that contextual fear memories created in the absence of the hippocampus seem to more heavily rely upon the PL (Zelikowsky et al., 2013). More research is required to further delineate the relationship between the PL and hippocampus in CFC.

### UFC Does Not Increase the Density of pMAPK, BDNF and IBA-1 Expressing Cells

Contextual fear memories formed in the presence of an unpaired auditory stimulus (UFC) produced a separate pattern of pMAPK, BDNF, and IBA-1 expression in the PL. As compared to the CO control, no noticeable difference could be identified. Moreover, BDNF expression and IBA-1 labelled microglia number were both found to be significantly lower in the UFC group as compared to the CFC group. The UFC protocol has typically been used as a control for auditory cue fear conditioning. This is because the auditory tone CS and foot-shock US are not paired, leading to the hypothesis that no associative memories are formed (McKernan and Shinnick-Gallagher, 1997; Rogan et al., 1997; Majak and Pitkänen, 2003; Radley et al., 2006; Bergstrom et al., 2011, 2013). While no associated auditory tone and foot-shock memories are formed, the ability for UFC to create associative fear memories to context is well documented (Phillips and LeDoux, 1994; Desmedt et al., 1998; Calandreau et al., 2005, 2006; Trifilieff et al., 2006, 2007). Furthermore, some research from our, and another, laboratory have demonstrated a statistically significant increase in the amygdala activity and plasticity following UFC (Trifilieff et al., 2007; Chaaya et al., 2019). In both cases, UFC appeared to cause larger increases in amygdala as compared to the CFC group (Chaaya et al., 2019) or the auditory cue fear condition group (Trifilieff et al., 2007). The data reported here, therefore, appears to contradict our previous research. Furthermore, the data reported here contradict previous research exploring the relative contribution of mPFC in trace fear conditioning. Trace fear conditioning is a more complex Pavlovian fear conditioning protocol whereby auditory tones and foot-shocks are presented at different (but consistently equal) temporal points (e.g., 20 s between every auditory tone and foot-shock presentation; Gilmartin et al., 2014). The complexity of this protocol requires additional circuitry, namely the PL and anterior cingulate cortex regions of the mPFC (Gilmartin and McEchron, 2005; Darling et al., 2011; Siegel et al., 2012; Gilmartin et al., 2014). While our results appear to be contradictory (with these trace fear conditioning studies and our past research), methodological differences can provide some clarity. Importantly, the UFC differs greatly from the trace fear conditioning protocol, as the random temporal presentation of auditory tone and foot-shock means no true “trace period” exists. That is, the tone does not predict the foot-shock. Consequently, the complexity and unpredictability of the UFC protocol may drastically alter the requirement for the brain regions identified as essential for trace fear conditioning. Further research into the maintenance of contextual fear memories formed with the UFC protocol is required.

### Evaluation of IBA-1 in Contextual Fear Memory Maintenance

This study investigated the alterations in microglia number and morphology 2 weeks following CFC and UFC. We demonstrate an increase in the number of IBA-1 microglia following CFC but fail to find any morphological alterations. The primary function of microglia is to respond to stimuli that harm central nervous system (CNS) function (Kettenmann et al., 2011; Calcia et al., 2016; Dwyer and Ross, 2016). Typically, this response involves an increase in the number of microglia to the affected area, as well as morphological alterations (Kettenmann et al., 2011; Calcia et al., 2016; Dwyer and Ross, 2016). The morphological alterations can be summarised as follows: when resting, microglia are ramified with smaller cell body, with many long thin extensions that search for signals of insult (Kettenmann et al., 2011; Walker et al., 2014; Dwyer and Ross, 2016). When responding to insult, microglia become amoeboid in shape, with extensions retracting and cell bodies enlarging. During this phase proinflammatory, immunoregulatory and other factors are released (Kettenmann et al., 2011; Walker et al., 2014; Dwyer and Ross, 2016).

Results in the current experiment showed an increase in microglia number following CFC. However, this increase in number was not accompanied by a change in the morphological state of microglia. While no other research to our knowledge has directly examined microglia morphology following Pavlovian fear conditioning, research into chemically induced (carbon dioxide) fear, as well as stress show that microglia alter morphology from a ramified resting phase to an active amoeboid state (Nair and Bonneau, 2006; Frank et al., 2007; Tynan et al., 2010; Calcia et al., 2016; Vollmer et al., 2016). Furthermore, recent research from our laboratory (Chaaya et al., 2019) demonstrates hippocampal microglia to alter morphology in a similar manner as a function of CFC. Therefore, two possible explanations for this data exist. First, microglial morphology may be altered in other brain regions which were not studied here, such as the amygdala or hippocampus. However, given the change in pMAPK, BDNF, and IBA-1 number shown here, this is not likely. Therefore, it is more likely that microglia morphology may have previously been active, but over time returned to a resting phase. This has been demonstrated previously (Jonas et al., 2012), with early research suggesting it may take up to 7 days to occur (McCann et al., 1996; Boucsein et al., 2000). Therefore, it appears that temporal factors did not allow for the morphological alterations of microglia to be assessed here.

### Technical Considerations

Evaluation of pMAPK, BDNF, and IBA-1 number in mPFC revealed a role for layers 2–3 of the PL following CFC, but not UFC. The particular experimental design utilised here allowed us to examine whether PL may be involved in the long-term maintenance of contextual fear memories. This advances previous knowledge which suggests this region is essential for the expression of contextual fears (see review by Rozeske et al., 2015 and research studies by Frankland et al., 2004; Corcoran and Quirk, 2007; Laurent and Westbrook, 2008; Goshen et al., 2011; Stevenson, 2011; Stern et al., 2013), but not the formation of contextual fears (Morgan et al., 1993; Morrow et al., 1999b; Fernandez Espejo, 2003; Antoniadis and McDonald, 2006; Bissière et al., 2008). However, the ability to accurately study fear expression vs. fear maintenance is difficult. Context is a broad term which encompasses elements, both internal and external to a person or animal (Maren et al., 2013). While anatomical rats in the current study did not undergo a fear memory test (meaning that fear expression did not occur, and was, therefore, not studied), fear memories may have been reactivated by other stimuli. For example, fear of the experimenter, or of other stimuli within the conditioning and housing units may have developed. We recommend that the daily handling of animals be performed in future experiments to remove this limitation. More importantly, fear memory generalisation (Jasnow et al., 2017) may have developed, so that fear towards the conditioning room may have generalised to fear of similar-looking rooms (e.g., the anaesthetic room). It is unlikely that the expression of this fear be significant enough to cause such large alterations in PL (suggesting that fear maintenance was truly studied here). Nevertheless, this is an important consideration given the level of complexity involved in memory processes.

The phrase “correlation does not imply causation” is commonly used to outline the inability for a cause-and-effect relationship to exist between variables based on their correlation. The current experiment manipulated an independent variable (CFC vs. UFC vs. CO control) to determine whether there are changes in expression, this does not allow for a causative relationship to be determined. However, a particular limitation is the inability to attribute these changes in expression to be directly involved in memory maintenance* per se*. That is, while we note changes occur in the mPFC 2 weeks following conditioning, we cannot show that memory is stored in layers 2–3 of the PL in the mPFC. More direct techniques that rely upon the direct manipulation of brain regions are required (Deisseroth, 2011; Roth, 2016). Unfortunately, these studies have their own limitations. Notably, inhibition of brain circuitry may have far-reaching consequences. For example, it becomes difficult to determine if inhibition of the mPFC disrupts memory maintenance or the ability of rodents to express fearful behaviour. This suggests a need for more intricately designed studies, whereby brain regions of interest are temporarily deactivated during a selected memory maintenance time-frame (e.g., for a 2 week period following CFC, but not during the memory recall period). Another potential experiment that could assess the functional role of these markers in fear maintenance would be to administer ANA-12, an antagonist for the BDNF receptor TrKB (Ma et al., 2020). If microinjection of ANA-12 into the pre-limbic cortex altered the maintenance of fear memory, then a functional role for BDNF could be confirmed.

BDNF is expressed by a variety of cell types in the rodent brain including neurons, microgla, astrocytes, and oligodendrocytes (Fletcher et al., 2018). While we have shown that CFC increases the number of BDNF positive cells within layers 2/3 of the PL, the cellular source of this change has not been identified. Although no overall net change in BDNF cell number was seen in layers 5/6 of the PL in response to CFC, there may still be alterations in the distribution of BDNF by cell type. Therefore, similar studies which co-stain BDNF with other brain cell-type markers (e.g., GFAP) would further elucidate the molecular mechanisms of long-term CFC maintenance. Additionally, the BDNF stained likely represents both intracellular BDNF as it is endogenously produced, as well as secreted BDNF bound to its receptor on neighbouring target cells. Without a counterstain of the nuclear marker 4′,6-diamidino-2-phenylindole (DAPI), it is not possible to distinguish between endogenous and secreted BDNF with the current data.

In addition to this, further research is required to examine whether the results reported here can be replicated in female rats. Research has identified various differences between male and female rats in cued fear conditioning. For example, female rats have been shown to have disruptions in the inhibition of fear (Toufexis et al., 2007). The authors noted differences arise due to higher levels of estrogen in female rats (Toufexis et al., 2007). Despite this, when contextual fear memories are examined, female rats seem to experience weaker fear memories. For example, CFC was recently found to be stronger in male rats when behavioural freezing was measured. This was similarly observed in male rats experiencing generalisation of fear to an open-field, while female rats did not (Daviu et al., 2014). Additional research into the sex differences of contextual fear found female rats to experience a less consistent and weaker renewal of fear, as compared to male rats (Anderson and Petrovich, 2015). Cumulatively, these results suggest that female rats experience weaker contextual fear memories compared to male rats. This difference seems to be specific to contextual fear memories, as opposed to cued fear memories (Toufexis et al., 2007). Therefore, further research is needed to extend our findings to determine whether comparable results would be obtained in female rats.

## Conclusion

The current study evaluated how contextual fear memory maintenance affected changes in the expression of MAPK, BDNF, and microglia within the PL of the mPFC by examining two different Pavlovian fear conditioning protocols. These protocols, both found to create equivalent fear memories to context, resulted in diverse mPFC alterations. We showed an increase in pMAPK expressing neurons, BDNF expressing cells, and IBA-1 labelled microglia as a function of CFC, but not UFC in PL layers 2–3, but not 5–6. While previous research exclusively studied fear expression, this study provides insights into the changes that occur in the mPFC 2 weeks following CFC. Despite the difference in IBA-1 number, we do not find a difference in the morphology of microglia. Further research is required to examine the interaction between microglia and neurons following long-term contextual fear memory maintenance.

## Data Availability Statement

The original contributions presented in the study are included in the article, further inquiries can be directed to the corresponding author.

## Ethics Statement

The animal study was reviewed and approved by Queensland University of Technology Animal Ethics Committee (approval number 80087) and University of Queensland Animal Ethics Committee (approval number 460/17).

## Author Contributions

NC, ARB, AJ, LRJ, FC, AB and SEB conceptualised and designed the study. NC and AJ performed all animal experiments. NC performed all immunohistochemistry and imaging. NC, MC, AB and JW analysed the data. NC created all figures. NC and JW wrote the manuscript. NC, AB, KB, JW and SEB revised the manuscript. All authors contributed to the article and approved the submitted version.

## Conflict of Interest

The authors declare that the research was conducted in the absence of any commercial or financial relationships that could be construed as a potential conflict of interest.
